# Genome-wide identification and analysis of *bZIP* gene family reveal their roles during development and drought stress in Wheel Wingnut (*Cyclocarya paliurus*)

**DOI:** 10.1186/s12864-022-08978-8

**Published:** 2022-11-08

**Authors:** Yu-Tian Tao, Lu-Xi Chen, Jie Jin, Zhao-Kui Du, Jun-Min Li

**Affiliations:** 1 School of Advances Study, Taizhou University, Taizhou, 318000 China; 2 School of Electronics and Information Engineering, Taizhou University, Taizhou, 318000 China; 3 Zhejiang Provincial Key Laboratory of Plant Evolutionary Ecology and Conservation, Taizhou University, Taizhou, 318000 China

**Keywords:** *Cyclocarya paliurus*, *bZIP* gene family, Leaf development, Drought stress, RT-qPCR

## Abstract

**Background:**

The *bZIP* gene family has important roles in various biological processes, including development and stress responses. However, little information about this gene family is available for Wheel Wingnut (*Cyclocarya paliurus*).

**Results:**

In this study, we identified 58 *bZIP* genes in the *C. paliurus* genome and analyzed phylogenetic relationships, chromosomal locations, gene structure, collinearity, and gene expression profiles. The 58 *bZIP* genes could be divided into 11 groups and were unevenly distributed among 16 *C. paliurus* chromosomes. An analysis of cis-regulatory elements indicated that *bZIP* promoters were associated with phytohormones and stress responses. The expression patterns of *bZIP* genes in leaves differed among developmental stages. In addition, several *bZIP* members were differentially expressed under drought stress. These expression patterns were verified by RT-qPCR.

**Conclusions:**

Our results provide insights into the evolutionary history of the *bZIP* gene family in *C. paliurus* and the function of these genes during leaf development and in the response to drought stress. In addition to basic genomic information, our results provide a theoretical basis for further studies aimed at improving growth and stress resistance in *C. paliurus,* an important medicinal plant.

**Supplementary Information:**

The online version contains supplementary material available at 10.1186/s12864-022-08978-8.

## Backgroud

The basic leucine zipper (*bZIP*) family, a supergene family encoding transcription factors (TFs), is evolutionarily conserved and widely distributed across eukaryotic organisms [1]. *bZIP* TFs contain a bZIP domain, generally composed of 60–80 amino acids, with two functionally distinct parts, a highly conserved basic region and a variable leucine-zipper region (explaining the name bZIP) [2, 3]. The basic binding region has a nuclear localization signal (NLS) and a N-X_7_-R/K structural unit [4, 5]. The *bZIP* gene family has been studied extensively in plants. The number of *bZIP* genes varies considerably among species, with 78 in *Arabidopsis* [1], 92 in rice [6], 86 in poplar [7], 50 in *Arachis duranensis* [8], and 52 in *Carthamus tinctorius* L. [9]. *bZIP* genes are involved in vital biological processes, including cell elongation, seed and flower development, and nitrogen/carbon and energy metabolism [10]. In addition to the essential regulatory functions in plant growth and development, *bZIP* genes participate in the response to abiotic stress. For instance, *bZIP17* and *bZIP24* in *Arabidopsis* [11, 12], *bZIP72* and *ABF1* in rice [13, 14], and *bZIP44*, *bZIP62*, and *bZIP78* in *Glycine max* [15] positively regulate plant responses to salt stress, either directly or indirectly. *bZIP52*, *bZIP16*, *bZIP23*, and *bZIP45* in rice are involved in drought tolerance [16–18]. Moreover, *bZIP52* in rice is a negative regulator in cold signaling [16]. *bZIP72* in rice positively regulates the ABA response [19], while *bZIP44*, *bZIP62*, and *bZIP78* in *G. max* show negatively regulatory effects [15].

*Cyclocarya paliurus* (Batal.) Iljinskaja (Wheel Wingnut), belonging to the family Juglandaceae [20], is a deciduous tree and is widely distributed in the mountainous regions of sub-tropical China [21]. In China, leaves of *C. paliurus* are used as a traditional medicine or nutraceutical tea [22]. Its leaves contain abundant physiologically active compounds [23], such as triterpenoids, polysaccharides, and flavonoids. Furthermore, there is evidence for strong health-promoting effects of its leaves, including the ability to lower blood sugar, reduce blood lipids, protect against cancer, and enhance immunity [24]. The growth and development of *C. paliurus* leaves are affected by environmental stress, such as drought, salt, cold, and heat [25], and various TFs contribute to the regulation of growth in *C. paliurus* leaves. For example, *bZIP* is involved in the regulation of amino acid biosynthesis [26], and *MYB* and *bHLH* are involved in the regulation of flavonoid biosynthesis [27]. The analysis of transcriptome data of the leaves in *C. paliurus* revealed the *bZIP* gene family was one of the most abundant TFs in this organism that regulate leaf development [26]. In addition to participate in leaf development, *bZIP* gene family is regarded as important regulators in signaling and responses to drought stress [16–18]. However, *bZIP* gene family characteristics have not been evaluated by integrative genome and transcriptomic analyses in *C. paliurus*.

The complete genome of *C. paliurus* has been sequenced, and 46,292 protein-coding genes have been identified [24]. In this study, we performed the genome-wide identification of the *bZIP* gene family and explored the structural characteristics of *bZIP* genes. We also measured the differential expression of *bZIP* genes at four developmental stages and under four drought stress treatments. We explored the evolution of *bZIP* genes and its roles in leaf developmental process and under drought stress. Our results provide a basis for further analysis of the molecular basis of growth, development, and stress responses in *C. paliurus* leaves.

## Results

### Genome-wide identification of *bZIP* family members in *C. paliurus*

We identified 58 *bZIP* genes in the *C. paliurus* genome, named *CpbZIP1* to *CpbZIP58* according to their localization on the chromosomes (Table 1). The lengths of *CpbZIP* mRNA transcripts and protein sequences ranged from 399 bp to 4,116 bp (CDS sequences) and 132 amino acids (*CpbZIP8*) to 1,371 amino acids (*CpbZIP22*) (translated protein sequences). The average molecular weight of *CpbZIP* family members was 43.39 kDa. The average isoelectric point (pI) of *CpbZIP* genes was 4.78 (*CpbZIP11*) to 9.53 (*CpbZIP27*). A plot of the molecular weight with pI for each *CpbZIP* gene revealed that the majority of *CpbZIPs* clustered together, indicating that they have a similar properties (Fig. S1). The grand average of hydropathy index (GRAVY) values for *CpbZIP* members ranged from -0.968 to -0.301, suggesting that these proteins are hydrophilic. All of the *CpbZIP* genes were predicted to be located in the nucleus, consistent with the biological function of TFs.

**Table 1:** Nomenclature and characteristics of the putative basic leucine zipper (bZIP) proteins in *C. paliurus*

Proposed Gene Name	Gene ID	Genomic Location	Group	CDS length (bp)	Protein Length (aa)	Molecular Weight (kDa)	Isoelectric Point (pI)	GRAVY	Predicted subcellular localization
*CpbZIP1*	*GWHPBEHY000088*	Chr01: 994,797–1,003,132	A	1,668	555	60.59	7.22	-0.302	Nucleus
*CpbZIP2*	*GWHPBEHY001972*	Chr01: 24,506,959–24,507,579	S	621	206	23.69	7.29	-0.804	Nucleus
*CpbZIP3*	*GWHPBEHY004160*	Chr02: 439,598–440,251	S	654	217	25.16	8.98	-0.968	Nucleus
*CpbZIP4*	*GWHPBEHY005548*	Chr02: 13,209,182–13,212,986	G	1,314	437	46.99	7.23	-0.741	Nucleus
*CpbZIP5*	*GWHPBEHY005552*	Chr02: 13,283,000–13,286,833	G	1,290	429	46.15	7.68	-0.788	Nucleus
*CpbZIP6*	*GWHPBEHY009574*	Chr02: 61,166,874–61,170,387	I	1,059	352	39.44	6.29	-0.709	Nucleus
*CpbZIP7*	*GWHPBEHY009820*	Chr03: 2,091,505–2,093,811	C	1,041	346	37.59	5.00	-0.488	Nucleus
*CpbZIP8*	*GWHPBEHY010183*	Chr03: 6,108,011–6,108,409	H	399	132	15.28	8.65	-0.677	Nucleus
*CpbZIP9*	*GWHPBEHY010213*	Chr03: 6,463,279–6,465,483	I	1,554	517	56.78	5.55	-0.740	Nucleus
*CpbZIP10*	*GWHPBEHY010283*	Chr03: 7,074,975–7,076,579	A	996	331	36.80	8.84	-0.360	Nucleus
*CpbZIP11*	*GWHPBEHY010475*	Chr03: 8,641,219–8,643,341	K	912	303	33.76	4.78	-0.467	Nucleus
*CpbZIP12*	*GWHPBEHY011792*	Chr03: 22,656,275–22,660,159	C	1,002	333	36.46	5.08	-0.867	Nucleus
*CpbZIP13*	*GWHPBEHY011812*	Chr03: 22,894,538–22,899,095	C	1,377	458	49.47	5.32	-0.753	Nucleus
*CpbZIP14*	*GWHPBEHY011961*	Chr03: 24,496,682–24,497,748	A	696	231	26.24	8.12	-0.408	Nucleus
*CpbZIP15*	*GWHPBEHY012394*	Chr03: 26,946,770–26,947,222	H	453	150	17.02	6.28	-0.675	Nucleus
*CpbZIP16*	*GWHPBEHY017071*	Chr04: 32,605,904–32,610,064	B	2,349	782	84.42	5.93	-0.460	Nucleus
*CpbZIP17*	*GWHPBEHY017112*	Chr04: 32,882,403–32,888,345	D	1,509	502	55.48	5.45	-0.517	Nucleus
*CpbZIP18*	*GWHPBEHY017380*	Chr04: 34,526,769–34,532,759	D	1,344	447	50.48	6.79	-0.687	Nucleus
*CpbZIP19*	*GWHPBEHY017617*	Chr05: 847,334–849,327	A	1,251	416	44.85	7.85	-0.533	Nucleus
*CpbZIP20*	*GWHPBEHY017951*	Chr05: 4,125,825–4,129,365	A	888	295	32.34	6.28	-0.662	Nucleus
*CpbZIP21*	*GWHPBEHY018195*	Chr05: 6,377,130–6,377,600	H	471	156	17.43	7.33	-0.474	Nucleus
*CpbZIP22*	*GWHPBEHY018243*	Chr05: 6,861,500–6,872,183	I	4,116	1,371	151.88	6.33	-0.510	Nucleus
*CpbZIP23*	*GWHPBEHY018315*	Chr05: 7,491,908–7,493,003	F	900	299	32.91	5.39	-0.499	Nucleus
*CpbZIP24*	*GWHPBEHY018692*	Chr05: 11,574,685–11,575,188	S	504	167	19.62	6.05	-0.948	Nucleus
*CpbZIP25*	*GWHPBEHY021408*	Chr05: 41,485,191–41,488,624	D	1,086	361	40.69	6.62	-0.484	Nucleus
*CpbZIP26*	*GWHPBEHY021409*	Chr05: 41,496,649–41,504,793	A	924	307	34.70	7.23	-0.455	Nucleus
*CpbZIP27*	*GWHPBEHY024306*	Chr06: 33,005,138–33,009,702	G	708	235	26.47	9.53	-0.942	Nucleus
*CpbZIP28*	*GWHPBEHY025069*	Chr06: 41,919,023–41,920,834	E	957	318	35.08	6.18	-0.652	Nucleus
*CpbZIP29*	*GWHPBEHY028595*	Chr07: 29,974,813–29,977,978	B	2,595	864	94.99	7.68	-0.466	Nucleus
*CpbZIP30*	*GWHPBEHY028621*	Chr07: 30,201,808–30,210,020	D	1,383	460	50.84	6.25	-0.563	Nucleus
*CpbZIP31*	*GWHPBEHY029014*	Chr08: 2,651,883–2,656,100	C	1,359	452	48.75	5.19	-0.637	Nucleus
*CpbZIP32*	*GWHPBEHY031317*	Chr08: 30,344,410–30,354,480	D	1,104	367	41.60	5.80	-0.449	Nucleus
*CpbZIP33*	*GWHPBEHY031880*	Chr09: 145,928–150,435	E	1,371	456	51.19	7.79	-0.741	Nucleus
*CpbZIP34*	*GWHPBEHY032467*	Chr09: 5,883,134–5,883,730	S	597	198	22.83	5.46	-0.857	Nucleus
*CpbZIP35*	*GWHPBEHY033340*	Chr09: 14,432,966–14,438,345	D	1,257	418	47.19	6.25	-0.301	Nucleus
*CpbZIP36*	*GWHPBEHY034341*	Chr09: 24,011,411–24,013,541	E	966	321	36.14	5.43	-0.873	Nucleus
*CpbZIP37*	*GWHPBEHY035033*	Chr10: 572,043–575,882	D	1,392	463	51.49	5.92	-0.333	Nucleus
*CpbZIP38*	*GWHPBEHY035146*	Chr10: 1,682,996–1,684,115	I	858	285	31.19	8.28	-0.538	Nucleus
*CpbZIP39*	*GWHPBEHY035968*	Chr10: 6,958,193–6,963,073	A	1,341	446	48.59	8.62	-0.554	Nucleus
*CpbZIP40*	*GWHPBEHY035973*	Chr10: 6,974,416–6,985,398	A	2,385	794	87.24	6.80	-0.404	Nucleus
*CpbZIP41*	*GWHPBEHY035975*	Chr10: 6,980,135–6,985,438	A	1,578	525	57.62	8.72	-0.731	Nucleus
*CpbZIP42*	*GWHPBEHY038013*	Chr11: 3,495,809–3,503,252	D	1,593	530	59.12	6.26	-0.412	Nucleus
*CpbZIP43*	*GWHPBEHY038367*	Chr11: 8,479,763–8,482,891	A	804	267	29.56	7.51	-0.824	Nucleus
*CpbZIP44*	*GWHPBEHY038368*	Chr11: 8,481,990–8,482,906	A	828	275	31.04	8.17	-0.568	Nucleus
*CpbZIP45*	*GWHPBEHY039260*	Chr11: 15,952,313–15,956,161	I	1,365	454	49.49	5.86	-0.602	Nucleus
*CpbZIP46*	*GWHPBEHY039600*	Chr11: 17,721,343–17,724,747	A	1,059	352	39.35	7.98	-0.700	Nucleus
*CpbZIP47*	*GWHPBEHY040320*	Chr11: 22,157,517–22,164,475	S	1,314	437	48.57	8.21	-0.440	Nucleus
*CpbZIP48*	*GWHPBEHY040408*	Chr11: 22,749,325–22,751,704	F	915	304	33.65	5.58	-0.520	Nucleus
*CpbZIP49*	*GWHPBEHY043047*	Chr12: 25,054,911–25,058,101	I	1,287	428	46.67	5.75	-0.651	Nucleus
*CpbZIP50*	*GWHPBEHY043240*	Chr12: 27,099,469–27,100,317	A	849	282	31.35	5.82	-0.731	Nucleus
*CpbZIP51*	*GWHPBEHY043284*	Chr12: 27,501,979–27,505,925	E	924	307	34.17	7.54	-0.619	Nucleus
*CpbZIP52*	*GWHPBEHY043579*	Chr12: 30,467,364–30,474,038	D	1,317	438	48.56	6.13	-0.462	Nucleus
*CpbZIP53*	*GWHPBEHY046497*	Chr15: 3,154,064–3,156,556	E	1,137	378	42.68	6.37	-0.967	Nucleus
*CpbZIP54*	*GWHPBEHY046600*	Chr15: 4,251,322–4,251,798	H	477	158	17.62	7.88	-0.504	Nucleus
*CpbZIP55*	*GWHPBEHY046675*	Chr15: 5,013,453–5,016,396	I	1,716	571	62.17	6.62	-0.827	Nucleus
*CpbZIP56*	*GWHPBEHY046757*	Chr15: 5,662,618–5,664,166	F	975	324	35.70	5.86	-0.733	Nucleus
*CpbZIP57*	*GWHPBEHY047010*	Chr15: 8,444,623–8,445,237	S	615	204	23.73	5.02	-0.867	Nucleus
*CpbZIP58*	*GWHPBEHY050133*	Chr16: 21,457,004–21,460,361	E	969	322	35.84	6.90	-0.827	Nucleus

**Fig. 1:**
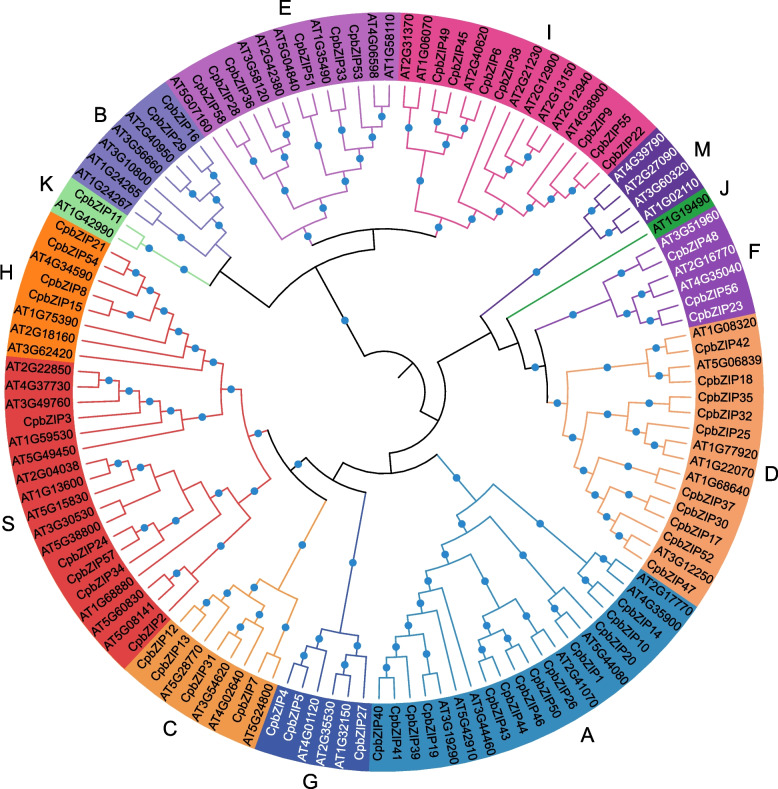
Phylogenetic analysis of CpbZIP proteins of *C. paliurus* and *Arabidopsis* using IQ-tree by the maximum likelihood method. Different groups are marked with different colors

To explore evolutionary relationships, we constructed a maximum likelihood phylogenetic tree based on the full-length sequences of proteins encoded by *bZIP* genes in *C. paliurus* and *Arabidopsis* (Fig. 1). The *bZIP* family members in *C. paliurus* and *Arabidopsis* were assigned to 13 groups according to the classification system for *Arabidopsis*. Only the bZIP proteins of *Arabidopsis* were assigned to group J and M. The three largest groups in *C. paliurus* included 13 (group A), 10 (group D), 7 (group I) CpbZIP members (Fig. S1 and Fig. S2).

### Chromosome localization, selective pressure, and collinearity analysis of *CpbZIP* genes

**Fig. 2:**
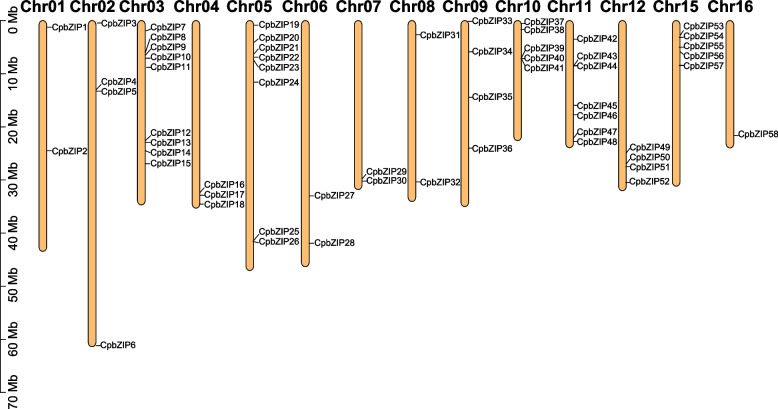
Chromosomal distribution of *CpbZIP* genes in *C. paliurus*. *CpbZIP* genes are marked at their approximate positions on the right side of chromosomes. The chromosome numbers are shown above each bar

All *CpbZIP* genes were found on 14 chromosomes of *C. paliurus* (Fig. 2 and Table 1), with an uneven distribution and substantial variation. Apart from Chromosome 13 and 14, which had no *CpbZIP* genes, chromosome 3 harbored the largest number of *CpbZIP* genes (9, 15.5%), while the fewest *CpbZIP* genes were detected on chromosome 16 (1, 1.7%). In addition, most of the *CpbZIP* genes were located near the ends of chromosomes.

Furthermore, we examined duplication events of *CpbZIP* family members. Based on the phylogenetic tree constructed (Fig. S3), several duplication events were predicted. In a survey of *CpbZIP* genes in the *C. paliurus* genome, 15 segmental duplications and 5 tandem duplications were identified, as shown in Figure S4 and Table S1, indicating that segmental duplication might play an important role in *bZIP* gene family expansion. Duplications of *CpbZIP* genes may have occurred at two time points, approximately 0.25–38.29 Mya and 80.60–99.47 Mya (Table S1). The non-synonymous substitution rate (*K*_a_), synonymous substitution rate (*K*_s_), and *K*_a_/*K*_s_ ratio for 21 duplicated gene pairs were calculated to evaluate selective pressure (Table S1). Values of *K*_a_/*K*_s_ < 1, *K*_a_/*K*_s_ = 1, and *K*_a_/*K*_s_ > 1 suggest purifying selection, neutral selection, and positive selection, respectively [28]. The *K*_a_/*K*_s_ ratios for all *bZIP* genes in *C. paliurus* were 0.1121–1.1166, and only one pair had a *K*_a_/*K*_s_ ratio exceeding 1.0, suggesting that most *CpbZIP* genes were under purifying selection.

**Fig. 3:**
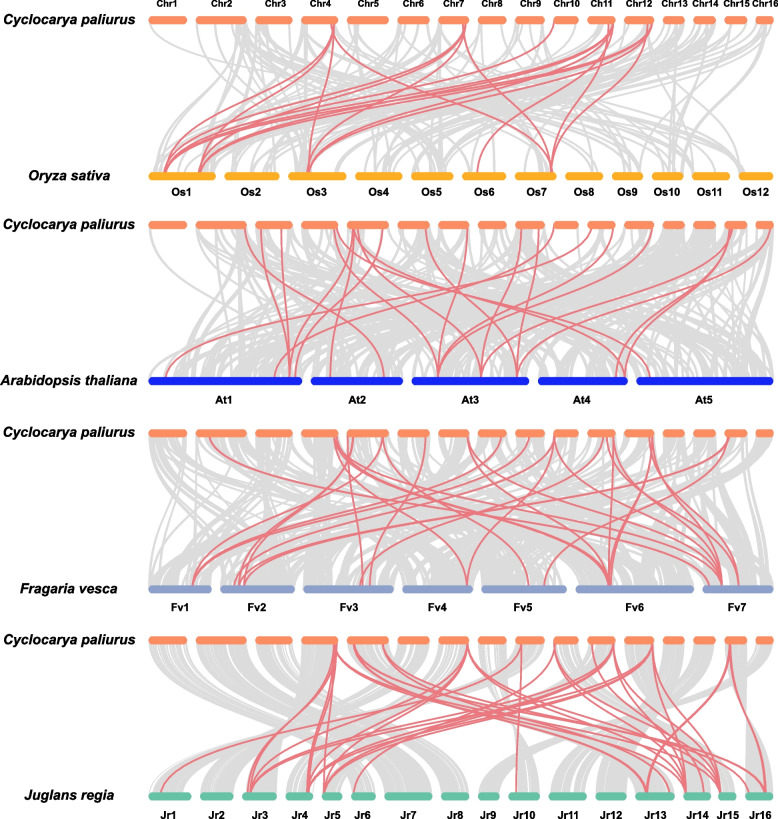
Syntenic relationships of *CpbZIP* genes between *C. paliurus* and *Oryza sativa*, *Arabidopsis thaliana*, *Fragaria vesca*, and *Juglans regia*. Gray lines in the background represent collinear blocks within *C. paliurus* and other plant genomes, while red lines highlight syntenic *bZIP* gene pairs

The collinearity between *C. paliurus bZIP* genes and related genes from four other species (i.e., *Oryza sativa*, *Arabidopsis thaliana*, *Fragaria vesca*, and *Juglans regia*) was also evaluated using the Multiple Collinearity Scan toolkit. In total, 33 *bZIP* genes in *C. paliurus* showed collinear relationships with 5 *O. sativa* genes, 12 *Arabidopsis* genes, 15 *F. vesca* genes, and 17 J*. regia* genes (Fig. 3 and Table S2). The numbers of orthologous gene pairs were 18 between *C. paliurus* and *O. sativa*, 22 between *C. paliurus* and *Arabidopsis*, 30 between *C. paliurus* and *F. vesca*, and 38 between *C. paliurus* and *J. regia*. Less orthologous gene pairs were found between *C. paliurus* and *O. sativa*, which may be explained by the closer phylogenetic relationships between *C. paliurus* and other species [24].

### Analyses of gene structure and conserved motifs

**Fig. 4:**
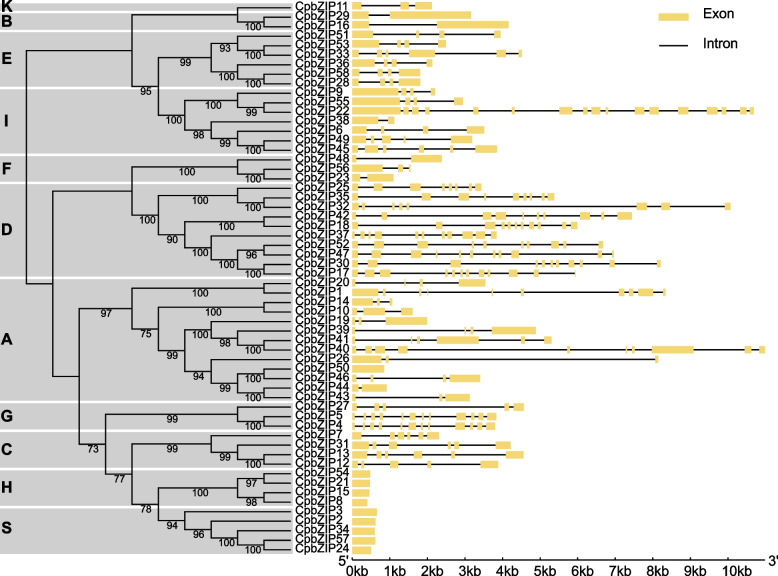
DNA structures of the *bZIP* gene family in *C. paliurus*. Exons are indicated by yellow bars and introns are denoted by black lines

**Fig. 5:**
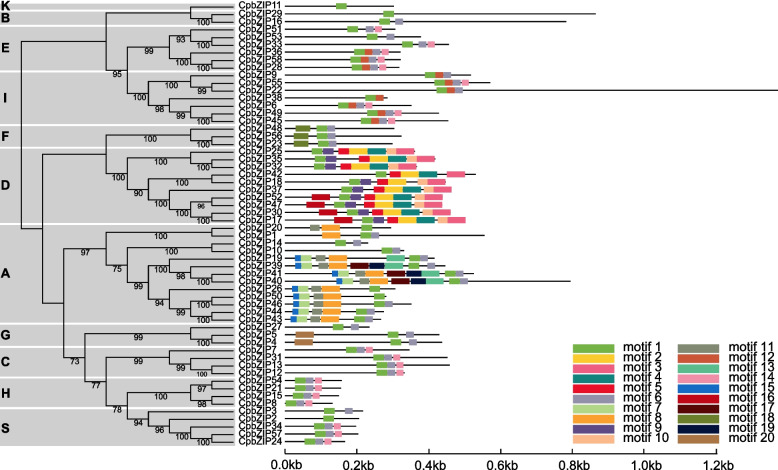
Protein motifs of the *bZIP* gene family members in *C. paliurus*. Box colors indicate different motifs. Clustering was performed according to the results of the phylogenetic analysis

To understand the sequence structure of the *bZIP* family in *C. paliurus*, the intron–exon structure (Fig. 4) and motif composition of each member (Fig. 5) were analyzed. *CpbZIP* genes had 1 to 17 exons. Most *CpbZIP* genes contained 1–3 introns, and some members of the *CpbZIP* gene family were intron-less, such as *CpbZIP2, CpbZIP3, CpbZIP8, CpbZIP15*, *CpbZIP21*, *CpbZIP24*, *CpbZIP34*, *CpbZIP50*, *CpbZIP54*, and *CpbZIP57*. A maximum of 16 introns were found in *CpbZIP22* (Fig. S5). Moreover, some *CpbZIP* members belonging to the same group shared similar gene structures (Fig. 4). For example, all members of group S and group H lacked introns. Out of six members in group E, five had four exons and three introns. Of four members in group C, three had six exons and five introns.

To discover conserved motifs of *CpbZIP* genes, we used MEME (Multiple Em for Motif Elicitation). A total of 20 conserved motifs were identified in 58 *CpbZIP* genes (Fig. 5), all of which had a bZIP domain (PF00170) represented by motif 1 (Table S3). Motif 6 and motif 14 were detected in the majority of *CpbZIP* members. In addition, motif 7, motif 8, and motif 15 occurred only in group A. Motif 12 was present only in group E and group I. Motif 2, motif 3, motif 4, motif 5, and motif 10 were located only in group A. Motif 18 was shared only by three members in group F. Many conserved motifs were found in specific groups and might be related to specific biological functions.

### Promoter region analysis of *CpbZIP* genes

**Fig. 6:**
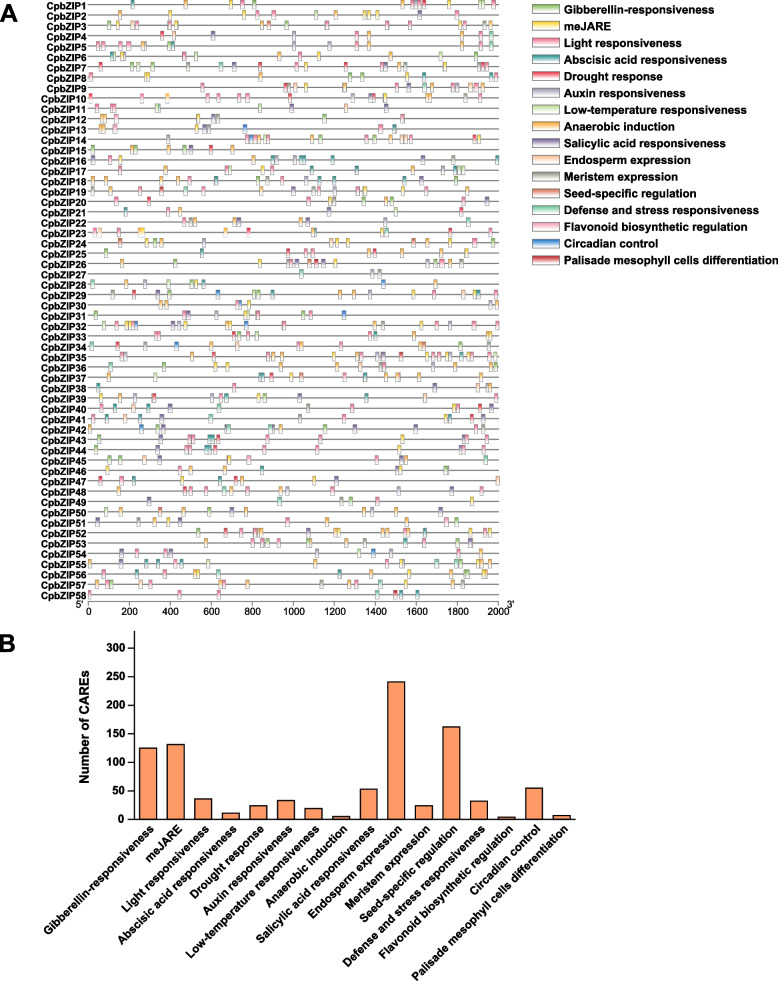
Putative cis-acting components of *bZIP* gene families in *C. paliurus*. **A** The promoter regions located 2000 bp upstream of the each *CpbZIP* gene were evaluated by a CARE analysis. Plant hormone response-related elements, stress response elements, light response elements, etc. are shown by different colors. **B** Number of each CARE in *CpbZIP* genes

We analyzed the 2000 bp region upstream of *CpbZIP* genes to elucidate cis-acting regulatory elements (CAREs) involved in processes related to development and the stress response using the PlantCARE webserver (Fig. 6). We found 16 unique CAREs in the *CpbZIP* gene family, including elements related to light responsiveness, defense and stress responsiveness, drought response, flavonoid biosynthetic regulation, and phytohormone responsiveness, including methyl jasmonate (meJA), gibberellin, abscisic acid, auxin, and salicylic acid. CAREs involved in light, plant hormone, and stress responses were most frequent in the *CpbZIP* gene family (Table S4 and Fig. 6B), suggesting that these genes are important for the regulation of plant growth and stress responses. Moreover, CAREs in *CpbZIP* members were also related to seed-specific regulation, meristem expression, and endosperm expression, indicating that these genes may be involved in diverse developmental processes. These data provide useful insights into the regulatory effects of the *CpbZIP* gene family under stress and during development.

### Gene ontology analysis of *CpbZIP* genes

To understand the functions of *bZIP* family members, we performed a Gene Ontology (GO) analysis [29–32]. *CpbZIP* genes were effectively annotated using eggNOG-Mapper (Table S5) [33]. In the biological process category, *CpbZIP* genes were enriched for processes related to phytohormones and stress responses (Fig. S6 and Table S6). The GO terms related to hormone responses included response to abscisic acid (GO:0,009,737), cellular response to hormone stimulus (GO:0,032,870), and abscisic acid-activated signaling pathway (GO:0,009,738). The GO terms related to the stress response included response to stimulus (GO:0,050,896), response to osmotic stress (GO:0,006,970), and response to salt stress (GO:0,009,651). The results of the GO analysis also further supported the roles of *CpbZIP* genes in biological processes related to plant development and stress responses.

### Expression of *CpbZIP* genes under drought stress and across developmental stages

**Fig. 7:**
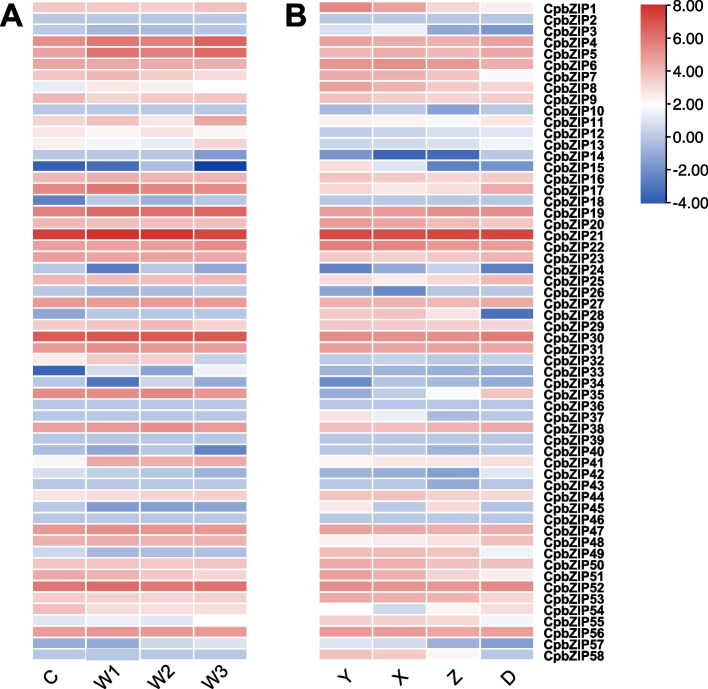
Heatmap representing the expression patterns of *CpbZIP* genes under drought stress (**A**) and across leaf developmental stages (**B**). Log_2_(FPKM) values were used to create the heatmap

To explore the expression pattern of *CpbZIP* genes at various leaf developmental stages and under drought stress, we retrieved fragments per kilobase million (FPKM) values for all *CpbZIP* genes from RNA-Seq data. We used FPKM values to build a principal component analysis (PCA) plot (Fig. S7) and heatmaps (Fig. 7). Four stages of leaf development and four drought treatments were evaluated (for details, please refer to the Materials and Methods section). Under drought treatment, Compared to the control C group of drought treatment, 361 different expressed genes (DEGs) were identified from W1 group, 427 DEGs were from W2 group, and 1,213 DEGs were from W3 group. Of 58 *CpbZIP* genes, 50 were expressed in the drought-treated samples (FPKM > 0) and showed differences in expression (Fig. 7A). For example, *CpbZIP4*, *CpbZIP5*, *CpbZIP19*, *CpbZIP22*, and *CpbZIP41* showed higher expression levels under drought stress condition (W1, W2, and W3) than in the control group (C). Moreover, during leaf development, 53 *CpbZIP* genes were expressed at different developmental stages, some of which showed higher expression in the smallest fully expanded leaves (Y stage) and small leaves (X stage) than in intermediate-sized leaves (Z stage) and in the largest fully expanded leaves (D stage) (Fig. 7B). *CpbZIP1*, *CpbZIP7*, *CpbZIP8*, *CpbZIP15*, *CpbZIP28*, *CpbZIP49*, *CpbZIP51*, and *CpbZIP55* were most highly expressed in the Y and X stages. These results indicated *CpbZIP* genes are important for drought tolerance and leaf development.

**Fig. 8:**
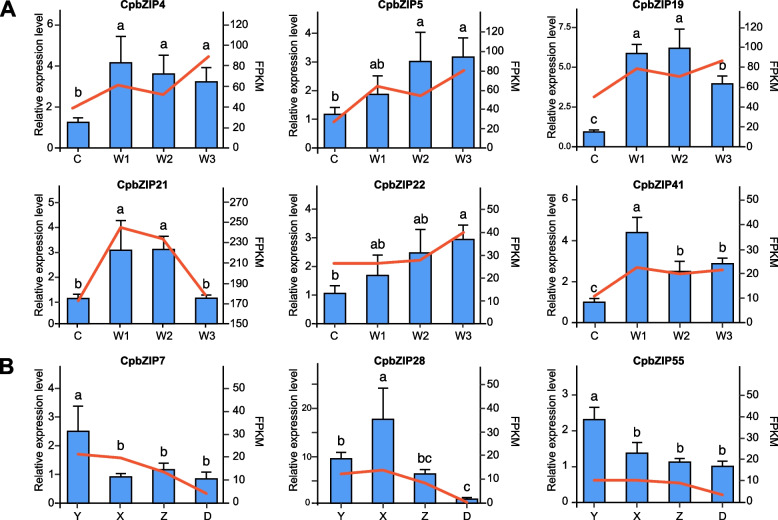
Quantitative real-time PCR analysis of nine *CpbZIP* genes in the response to drought stress (**A**) and leaf development (**B**) for the verification of RNA-Seq results. Actin of *C. paliurus* was used as the internal control for standardization. C: 22.5–25.5% soil water, W1: 16.5–19.5% soil water, W2: 10.5–13.5% soil water, and W3: 4.5–7.5% soil water. Y: smallest fully expanded leaves, X: small leaves, Z: intermediate-sized leaves, and D: the largest fully expanded leaves. Error bars indicate SD, and different lowercase letters (a–c) represent significant differences at *p* < 0.05

To confirm the RNA-Seq results, nine differentially expressed genes were selected for validation by qRT-PCR. As shown in Fig. 8A, all selected *CpbZIP* genes were up-regulated under drought stress. The expression levels of *CpbZIP4*, *CpbZIP19*, and *CpbZIP41* were significantly higher in all three drought treatments than in the control, while *CpbZIP5* expression was significantly higher in W2 and W3 conditions and *CpbZIP21* expression was highest in W1 and W2 conditions. An increase in the expression level of *CpbZIP22* was detected in W3. During leaf development, as shown in Fig. 8B, *CpbZIP7* and *CpbZIP55* were highly expressed in the Y developmental stage, while *CpbZIP28* was highly up-regulated in the X developmental stage.

### Co-expression analysis

**Fig. 9:**
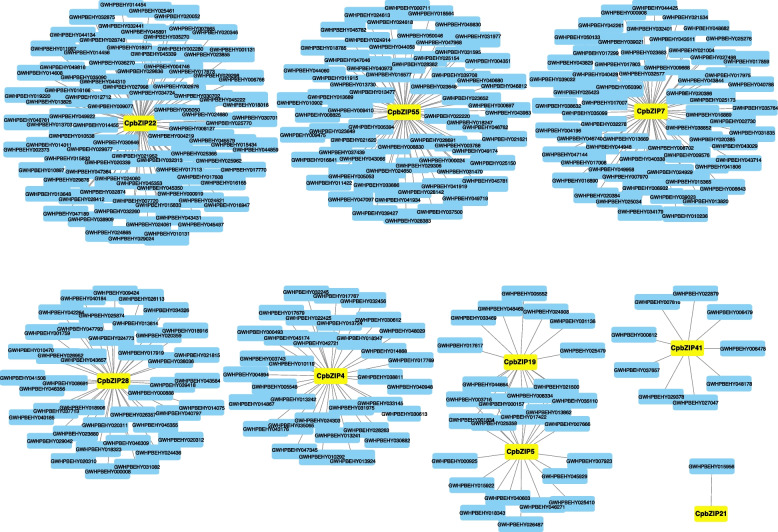
Co-expression network of five drought stress response-related *CpbZIP* genes and three development-related *CpbZIP* genes. Yellow rectangles represent *CpbZIP* genes and blue rectangles represent co-expressed genes. Grey lines indicate co-expression

Co-expression analysis is a powerful approach to screen associated genes, which may be co-regulated or involved in the same signaling pathway or physiological process [34]. Therefore, co-expression networks were constructed based on the differently expressed genes under developmental and drought stress conditions in *C. paliurus*. The nine genes with expression changes supported by both RNA-Seq and qRT-PCR (*CpbZIP4*, *CpbZIP5*, *CpbZIP7*, *CpbZIP19*, *CpbZIP21*, *CpbZIP22*, *CpbZIP28*, *CpbZIP41*, and *CpbZIP55*) and mRNAs from plant leaves were used to identify patterns of co-expression (Fig. 9). Nine co-expression networks were obtained, including 342 significantly correlated gene pairs. Among these, the network centered on *CpbZIP22* was the largest (90 genes). The network centered on *CpbZIP21* was the smallest, with only one co-expressed gene. In addition, with the annotation of 342 significantly correlated gene pairs, several genes were found to be involved in the responses to the water deprivation (Table S7).

**Fig. 10:**
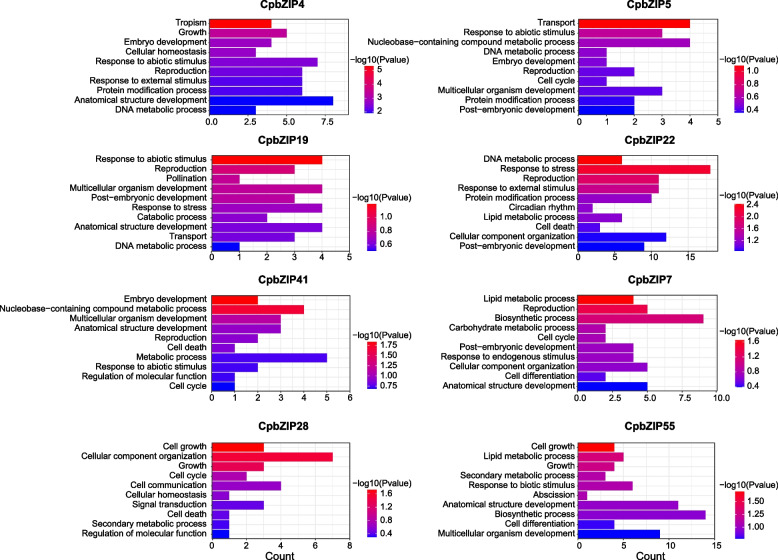
GO enrichment analysis of eight co-expressed gene sets

We performed a gene set enrichment analysis of eight sets of co-expressed genes (the smallest network involving *CpbZIP21* was excluded). The ten most significant GO terms were selected for each set (Fig. 10). *CpbZIP4*, *CpbZIP5*, *CpbZIP19*, *CpbZIP22*, and *CpbZIP41,* which were up-regulated under drought stress, were enriched for the response to abiotic stimulus (GO:0,009,607), response to external stimulus (GO:0,009,605), and response to stress (GO:0,006,950). In addition, *CpbZIP7*, *CpbZIP28*, and *CpbZIP55,* which were highly expressed in during leaf development (Y stage and X stage), were enriched for reproduction (GO:0,000,003), post-embryonic development (GO:0,009,791), and growth (GO:0,040,007). *CpbZIP* genes may therefore play important roles in the regulation of *C. paliurus* growth and development and stress responses.

## Discussion

*C. paliurus* is an endangered plant that only grows in China and is a very important medical plant; its leaves contain polysaccharides, triterpenoids, and other chemical components with numerous health benefits [23]. In plants, *bZIP* TFs have been reported to contribute to developmental processes and abiotic stress tolerance [35]. Members of the *bZIP* family have been comprehensively identified and analyzed in several plants, including *Arabidopsis* [1], rice [6], poplar [7], *Arachis duranensis* [8], and *Carthamus tinctorius* L. [9]. Although a chromosome-scale genome assembly of *C. paliurus* has been reported, *bZIP* genes have not been comprehensively identified and their roles in leaf development and drought stress are unclear. In this study, 58 *bZIP* genes were identified in the *C. paliurus* genome by a homology search. A transcriptome analysis of *C. paliurus* revealed 60 differentially expressed *bZIP* genes among different developmental stages [26], which was higher than number of genes identified in our genome-wide homology-based search. This may explained by the transcriptomic data obtained from four sub-genomes in autotetraploid *C. paliurus* and the lack of bZIP domain validation. In addition, compared to the genes predicted from transcriptomic data, genome-wide identification combined with a transcriptomic analysis can provide more information on gene structures, functions, and expression patterns [36, 37]. Further chromosome-level assemblies of the four sub-genomes may facilitate more comprehensive functional studies of *bZIP* genes and their regulatory mechanisms in *C. paliurus*. The genomic survey revealed 58 members of the *C. paliurus bZIP* gene family, which was fewer than estimates in *Arabidopsis* (78 *bZIPs*), rice (92 *bZIPs*), maize (125 *bZIPs*), and poplar (86 *bZIPs*) [1, 6, 7, 38]. Similar to the *C. paliurus* family, the bZIP families in *Arachis duranensis* (50 *bZIPs*) and *Carthamus tinctorius* L (52 *bZIPs*) were relatively small [8, 9], indicating that the gene family in these taxa contracted during evolution.

In this study, all *CpbZIPs* were predicted to be located in the nucleus, consisting with the TF characteristics and experimental studies in other organisms, such as rice [39]. Moreover, the 58 *CpbZIP* genes were not uniformly distributed across the 16 chromosomes in *C. paliurus* (Fig. 2) and were preferentially located near the ends of the chromosomes, similar to observations in sweet potato [10], *Cucumis sativus* [40], and wheat [41]. Based on the phylogenetic reconstruction (Fig. 1), *bZIP* genes in this study could be categorized into 13 groups; *C. paliurus* lacked *CpbZIP* genes in group J and group M in *Arabidopsis,* suggesting that genes in these groups diverged or were lost in *C. paliurus* [42]. Recent studies have proposed that gene duplication events are the main driving forces for gene family expansion and genome evolution, particularly segmental duplication and tandem duplication [43, 44]. In the expansion of the *bZIP* gene family, segmental duplications are more common than tandem duplications in many plants, such as *Ipomoea trifida* [10], *Malus halliana* [45], and wheat [41]. We detected 15 gene pairs with evidence for segmental duplications and 5 pairs with evidence for tandem duplications (Table S1), consistent with these previous findings. Most *CpbZIPs* (95.24%) showed evidence for purifying selection (*K*_a_/*K*_s_ > 1) [28], indicating that *CpbZIP* genes in *C. paliurus* are highly conserved. One gene pair with *K*_a_/*K*_s_ above 1.0 may be under positive selection [46], with rapid recent evolution and potential functional importance [47]. Furthermore, that there was greater collinearity between *C. paliurus* and *J. regia* than between *C. paliurus* and other plants due to the relatively closer evolutionary relationships [24]. In *C. paliurus*, *CpbZIP* members showed similar gene structures in the majority of subfamilies (Fig. 4), especially in the number and length of exons, consistent with results reported in wheat [41]. A motif analysis (Fig. 5) revealed 20 motifs in *C. paliurus,* named motif 1 to motif 20 (Fig. 5), consistent with results in wheat [41], *Carthamus tinctorius* [9], and cassava [48]. In addition to the bZIP domain (motif 1) located in each *CpbZIP* gene, the overall compositions of motifs were similar within the same subgroup but different among groups, indicating that functional divergence of *bZIP* genes may be determined by group-specific motifs [8]. This was consistent with results of studies of polar [7] and *Malus halliana* [45]. Both gene structure and motif analyses support the classification of *bZIP* genes in the phylogenetic analysis.

Several studies have demonstrated the roles of plant bZIP proteins in numerous developmental processes and in responses to biotic and abiotic stresses [8, 49–52]. However, little is known about their functions in *C. paliurus*. In this study, we explored their expression patterns after drought stress treatment and during different stages of leaf development. A transcriptome analysis revealed that a large number of *CpbZIP* genes were up-regulated after drought treatment or in the Y stage and X stage (Figs. 7 and 8), such as *CpbZIP4*, *CpbZIP5*, *CpbZIP7*, *CpbZIP19*, *CpbZIP21*, *CpbZIP22*, *CpbZIP28*, *CpbZIP41*, and *CpbZIP55*, indicating *CpbZIPs* have vital functions in leaf development and responses to drought stress. Similarly, the cis-acting elements in promoter regions contained a variety of components involved in the stress response (drought response, low-temperature response, and defense and stress response) and phytohormone responses (gibberellin, auxin, abscisic acid, salicylic acid, and methyl jasmonate) (Fig. 6). These results supported the important roles of the *CpbZIP* gene family in environmental stress and plant development, consistent with previously reported functions of *bZIP* TFs [1, 4, 15–17, 19, 51]. In the present study, in addition to the up-regulated genes, some *CpbZIPs* were down-regulated in response to drought stress and during leaf development, indicating that *CpbZIP* TFs might act as positive or negative regulators. This phenomenon has been reported in other organisms. For example, *AtbZIP17* and *AtbZIP24* act as positive regulators in *Arabidopsis* under salt stress [11, 12], while *OsbZIP52* [16] in rice functions as a negative regulator in cold signaling. Moreover, *OsbZIP72* in rice positively regulates the ABA response [19], while *GmbZIP44* and *GmbZIP62* in *Glycine max* show negatively regulatory effects [15]. To understand *bZIP* gene functions in *C. paliurus*, co-expression network and gene set enrichment analyses were performed (Figs. 9 and 10). The differentially expressed genes at different developmental stages and their corresponding networks were mainly enriched in processes related to plant growth, while differentially expressed genes in drought stress were not only enriched in stress response-related biological processes but also in growth-related processes. These results suggested that *CpbZIP* genes are potentially involved in drought resistance and leaf development in *C. paliurus*. Nonetheless, further experimental analyses should be carried out to elucidate the precise regulatory mechanism by which *CpbZIP* genes contribute to the response to drought stress and development.

## Conclusions

*C. paliurus* is an endangered medical plant distributed in the mountainous regions of sub-tropical China. Research has mainly focused on increasing yield, quality, and stress tolerance in *C. paliurus*. The *bZIP* gene family is involved in plant growth and development and plays important roles in the tolerance to environmental stress. In this study, we identified and characterized the *bZIP* gene family in *C. paliurus*. Expression profiling and functional enrichment analyses clearly demonstrated the role of *CpbZIPs* in leaf development and the response to drought stress. The results of this study improve our understanding of the role of *bZIPs* in developmental processes and in drought stress and provide a good foundation for further studies of the molecular regulatory mechanisms underlying *C. paliurus* stress resistance and growth.

## Methods

### Genome-wide identification of *bZIP* transcription factors in *C. paliurus*

The hidden Markov model of the bZIP domain (PF00170) was obtained from the PFAM database (http://pfam.xfam.org/, accessed on 19 November 2021) and the genome sequence and genome annotation of *C. paliurus* were downloaded from Genome Warehouse in National Genomics Data Center Beijing Institute of Genomics, Chinese Academy of Sciences/China National Center for Bioinformation (https://ngdc.cncb.ac.cn/gwh, under accession number GWHBEHY00000000, accessed on 18 December 2021). To identify *CpbZIP* genes in *C. paliurus*, two methods were applied. First, a local database of protein sequences was made for *C. paliurus*, and *bZIP* genes from *Arabidopsis* were utilized to discover putative *bZIP* genes in *C. paliurus* by BLASTp searches. A cutoff e-value of 10^–5^ and bit score of 100 were thresholds for the identification of putative *bZIP* genes. Second, another protein sequence database of *bZIP* genes from other plant species was built from Ensembl hosts (http://plants.ensembl.org/index.html, accessed on 21 February 2022). Then, BLASTp searches were performed against the proteome of *C. paliurus* with an e-value threshold of 10^–5^ and bit score threshold of 100. After removing redundancy, 72 putative *bZIP* candidates were obtained, which were further verified for the existence of the bZIP domain (PF00170) using HMM-scan (https://www.ebi.ac.uk/Tools/hmmer/search/hmmscan), NCBI CDD (https://www.ncbi.nlm.nih.gov/Structure/cdd/cdd.shtml), interPro (https://www.ebi.ac.uk/interpro/), and SMART tools (https://smart.embl-heidelberg.de/). After removing sequences without bZIP domains, 58 *bZIP* genes were named according to the locations on the chromosomes.

### Sequence analysis of *CpbZIP* genes in *C. paliurus*

The isoelectric point and molecular weight of CpbZIP proteins were characterized using the isoelectric point calculator (https://web.expasy.org/compute_pi/). CELLO [53, 54] was used to predict the subcellular localization of CpbZIP proteins. The annotation file was utilized to extract intron–exon distributions and gene structures were visualized using Gene Structure Display Server 2.0 [55]. MEME [56] was used to elucidate conserved motifs. The maximum number of motifs was set to 10, motif width was 6–20, and other parameters were set to default values. For the identification of CAREs, the 2000 bp sequences upstream of the *CpbZIP* genes were analyzed by the PlantCARE online server (http://bioinformatics.psb.ugent.be/webtools/plantcare/html) and visualized using TBtools [57].

### Chromosomal location, gene duplication, and synteny analysis

The genomic positions of *CpbZIP* genes and length of each chromosome were extracted from genome sequence and annotation files using local Perl scripts. TBtools was used to represent *CpbZIP* genes on *C. paliurus* chromosomes. MCScanX was used to investigate gene duplication events within *C. paliurus* species and similarity between *bZIP* genes in *C. paliurus* and four species, *Oryza sativa*, *Arabidopsis thaliana*, *Fragaria vesca*, and *Juglans regia*. Data for the first three species were downloaded from the Phytozome database [58] and data for *Juglans regia* were downloaded from the NCBI Nucleotide database (NC_049901–NC_049916). The nonsynonymous substitution rate and synonymous substitution rates were calculated using DnaSP [59]. The time of each gene duplication event was calculated with formula T = *K*_s_/2λ, assuming 6.5 × 10^−9^ synonymous substitutions per site per year [41, 60, 61].

### Plant material and drought treatment

Leaf materials of *C. paliurus* were collected from ZhuZhang Village, Longquan City, Lishui City, Zhejiang province, China (E118°48’28”, N28°5’57”). Leaves were divided into four development stages, including the smallest fully expanded leaves (Y stage), small leaves (X stage), intermediate-sized leaves (Z stage), and the largest fully expanded leaves (D stage). The leaves of *C. paliurus* were sampled separately on the same tree at the same time of each developmental stage. The collected leaves were stored in a liquid nitrogen tank immediately after being collected from the branches. Then the leaves were transferred to -80℃ freezer for storage after returning to the laboratory. Three biological replicates were independently performed, and each developmental stage contained three plants in one biological replicate. To avoid experimental errors between repetitions, we collected leaves of four developmental stages on the same tree with different orientations at the same time. In addition, one replicate of each developmental stage mixed the leaves from three randomly selected trees. For each developmental stage, the whole leaves were used for further RNA-seq analysis.

For the drought treatment, 2-year-old *C. paliurus* seedlings were moved to greenhouse in Taizhou University with a ratio of peat soil to vermiculite of 2:1. After the seedlings were adapted to the growth environment and maintained stable growth, four drought treatments were applied for 100 days, including 22.5–25.5% soil water (control C group), 16.5–19.5% soil water (W1), 10.5–13.5% soil water (W2), and 4.5–7.5% soil water (W3). Similar to the developmental leaf materials, three biological replicates for each drought treatment were included for transcriptome analyses.

### Transcriptome analysis

Transcriptomic data for *C. paliurus* leaves at four developmental stages were collected as described previously by Sheng et al. [27] and were downloaded from the NCBI database with accession no. PRJNA548403. For different drought treatment groups, total RNA was extracted from the leaves using a Total RNA Extractor (TRIzol) Kit (B51311; Sangon Biotechnology, Shanghai, China). Three biological replicates were performed for a total of 12 samples, which were used for mRNA library construction after the determination of the quality and concentration of extracted RNAs using the NanoDrop 2000 (Thermo Fisher, Waltham, MA, USA). mRNA libraries were constructed using the VAHTS mRNA-seq V2 Library Prep Kit for Illumina (NR60102; Vazyme Biotechnology, Nanjing, China). The T100TM thermal cycler (Bio-Rad, Hercules, CA, USA) was used to synthesize the first- and second-strand cDNAs, and the library fragments were further purified by AMPure XP System (Beckman Coulter Company, Beverly, MA, USA). After library amplification by PCR, the products were purified using the AMPure XP system and qualified using the Bioanalyzer 2100 system (Agilent Technologies Inc., Santa Clara, CA, USA). Finally, paired-end sequencing of these libraries was performed using HiSeq X Ten sequencers (Illumina, San Diego, CA, USA) by Novagen Co., Ltd. (Beijing, China). After removing the adapters and low-quality reads using Trimmomatic [62], the trimmed reads were aligned to the *C. paliurus* genome using HISAT2 with default parameters [63]. The expression profiles including FPKM values and read counts for each *CpbZIP* gene were calculated using StringTie [64] with default parameters. Heatmaps and a principal component analysis (PCA) were performed using TBtools [57] and the FactoMineR R package [65].

### Real-time PCR analysis

RNAs extracted from plants at different developmental stages and under drought stress were treated with DNase-I (Takara Bio. Inc., Shiga, Japan) at 37 °C for 30 min to remove genomic DNA contamination. RNAs were reverse transcribed to cDNA using the cDNA Synthesis SuperMix Kit (Applied Biosystems, Shanghai, China). Quantitative real-time PCR (qRT-PCR) was performed using SYBR qPCR Master MIX (Vazyme). Three biological replicates were included for each sample. Relative expression by qRT-PCR was normalized to beta actin (β-actin). The fold change values were calculated based on mean 2^−∆∆CT^ values [41]. Primers were designed using the Sangon Biotech online server (https://www.sangon.com/newPrimerDesign). The primers are listed in Table S8.

### Gene co-expression and gene ontology analysis

Nine differentially expressed *CpbZIP* genes were evaluated. Co-expression between *CpbZIP* genes and non-*CpbZIP* genes was evaluated based on Pearson correlation coefficients (PCC). Gene pairs for which the absolute value of the PCC was higher than 0.99 (*p* < 0.01) were regarded as co-expressed. Cytoscape [66] was used for network visualization. A gene set enrichment analysis was performed using the clusterprofiler package in R [67].

## Supplementary Information

**Additional file 1:** **Fig. S1.** Molecular weight (kDa) vs. isoelectric point for *CpbZIP *genes.

**Additional file 2:** **Fig. S2.** Distribution of *CpbZIPs *in different groups in the phylogenetic tree.

**Additional file 3:** **Fig. S3.** Phylogenetic analysis of *CpbZIP *genes. The phylogenetic tree was constructed using IQ-tree with the maximum likelihood (ML) method and 1000 bootstrap replications. Black asterisks indicate putative duplicated genes.

**Additional file 4:** **Fig. S4.** Chromosomal distribution and duplicated *CpbZIP *gene pairs. Duplicated *bZIP *gene pairs are connected by lines with distinct colors.

**Additional file 5:** **Fig. S5.** Distribution of intron numbers in *CpbZIP *genes in different groups according to the phylogenetic tree.

**Additional file 6:** **Fig. S6.** Gene Ontology term distribution in *CpbZIP *genes.

**Additional file 7:** **Fig. S7.** PCA plots displaying differentiation with respect to developmental stages and drought stress conditions based on *CpbZIP *expression patterns.

**Additional file 8:** **Table S1.** Information on duplicated bZIP gene pairs in C. paliurus. **Table S2.** Orthologous relationships between CpbZIP genes and bZIP genes in Oryza sativa, Arabidopsis thaliana, Fragaria vesca, and Juglans regia. **Table S3.** Domain organization of CpbZIP genes predicted using pfam. **Table S4.** Cis-regulatory elements in CpbZIP promoter regions. **Table S5.** Gene annotation using eggnog-mapper. **Table S6.** Gene Ontology analysis of CpbZIP genes. **Table S7.** Potential genes involved in drought stress responses according to 342 significantly correlated gene pairs. **Table S8.** qRT-PCR primers for CpbZIP genes.

## Data Availability

The raw RNA-Seq data of drought treatment groups in *C. paliurus* analyzed in this study have been deposited in the Nation Center for Biotechnology Information (NCBI) Sequence Read Archive (SRA) database under the accession number PRJNA870281. The transcriptomic data for *C. paliuru*s leaves at four developmental stages that analyzed in this study were from the NCBI database with accession number PRJNA548403. The genome sequence and genome annotation of *C. paliurus* were from Genome Warehouse in National Genomics Data Center Beijing Institute of Genomics, Chinese Academy of Sciences/China National Center for Bioinformation (https://ngdc.cncb.ac.cn/gwh, accession number GWHBEHY00000000).

## Abbreviations

bp: Base pair
TF: Transcriptional factor
bZIP: Basic leucine zipper
RT-qPCR: Real time quantitative polymerase chain reaction
pI: Isoelectric point
GRAVY: Grand average of hydropathy index
NLS: Nuclear localization signal
Mya: Millions of Years Ago
*K*_a_: Non-synonymous substitution rate
*K*_*s*_: Synonymous substitution rate
CARE: Cis-acting regulatory elements
meJA: Methyl jasmonate
PCA: Principal component analysis
