# Supplementary figures and images for: Genome-wide identification and analysis of bZIP gene family reveal their roles during development and drought stress in Wheel Wingnut (Cyclocarya paliurus)
Source: BMC Genomics. 2022 Nov 8;23:743. doi: 10.1186/s12864-022-08978-8 (PMC9641814; doi:10.1186/s12864-022-08978-8)

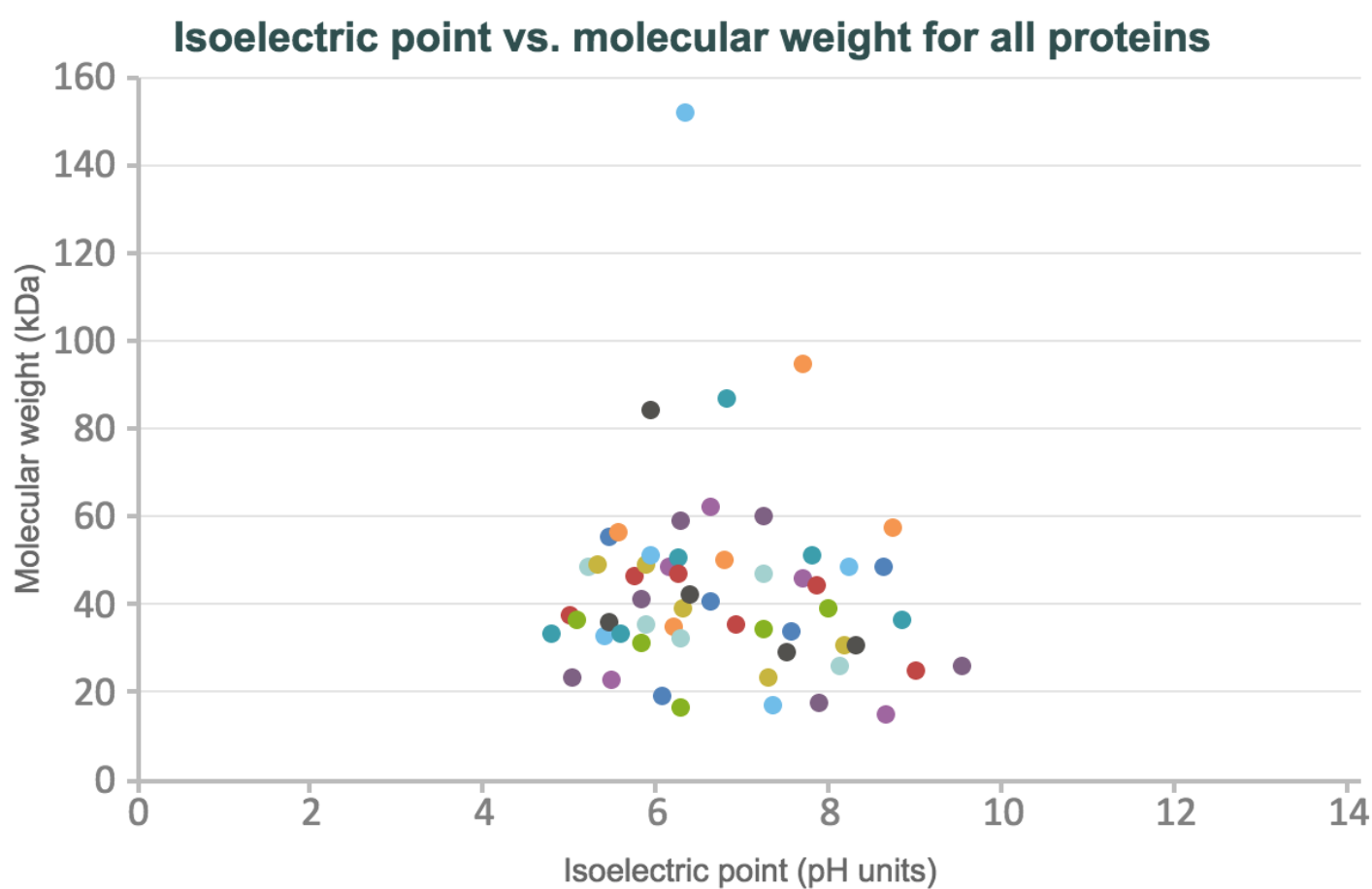

Fig. S1. Molecular weight (kDa) vs. isoelectric point for *CpbZIP* genes.

Supplement: Supplementary file 1 — Additional file 1: Fig. S1. Molecular weight (kDa) vs. isoelectric point for CpbZIP genes. [file 12864_2022_8978_MOESM1_ESM.pdf]

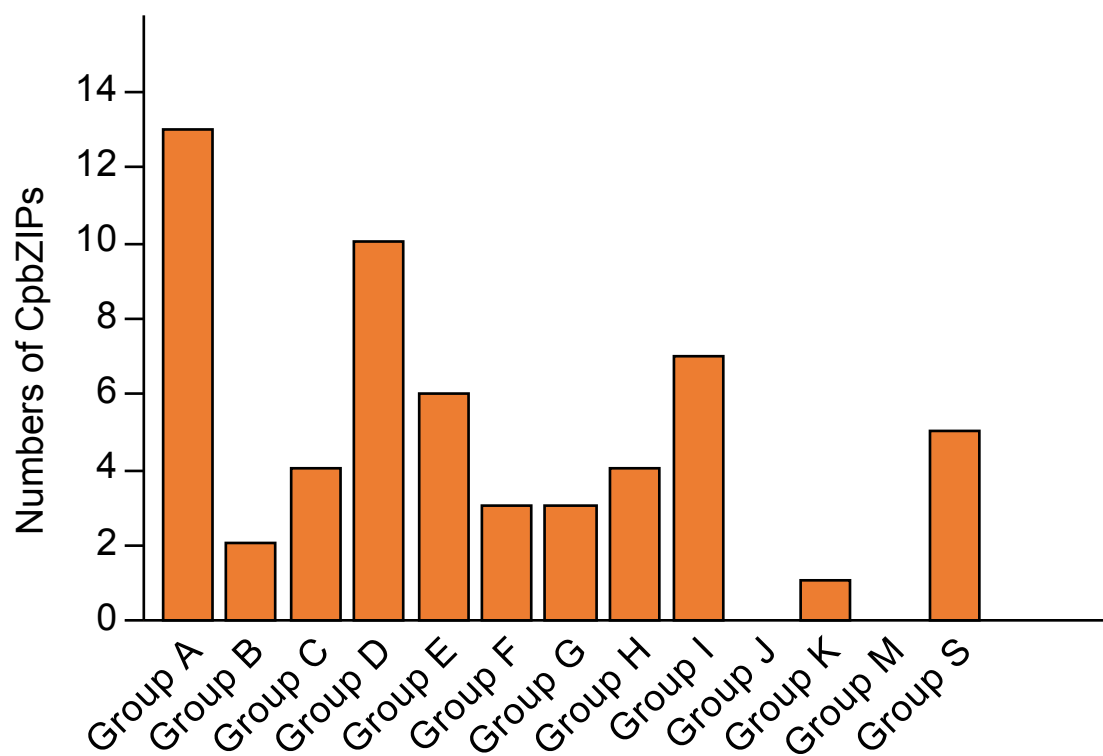

Fig. S2. Distribution of *CpbZIPs* in different groups in the phylogenetic tree.

Supplement: Supplementary file 2 — Additional file 2: Fig. S2. Distribution of CpbZIPs in different groups in the phylogenetic tree. [file 12864_2022_8978_MOESM2_ESM.pdf]

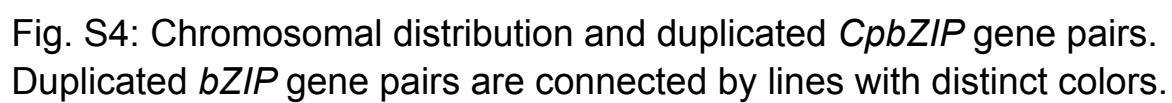

Supplement: Supplementary file 4 — Additional file 4: Fig. S4. Chromosomal distribution and duplicated CpbZIP gene pairs. Duplicated bZIP gene pairs are connected by lines with distinct colors. [file 12864_2022_8978_MOESM4_ESM.pdf]

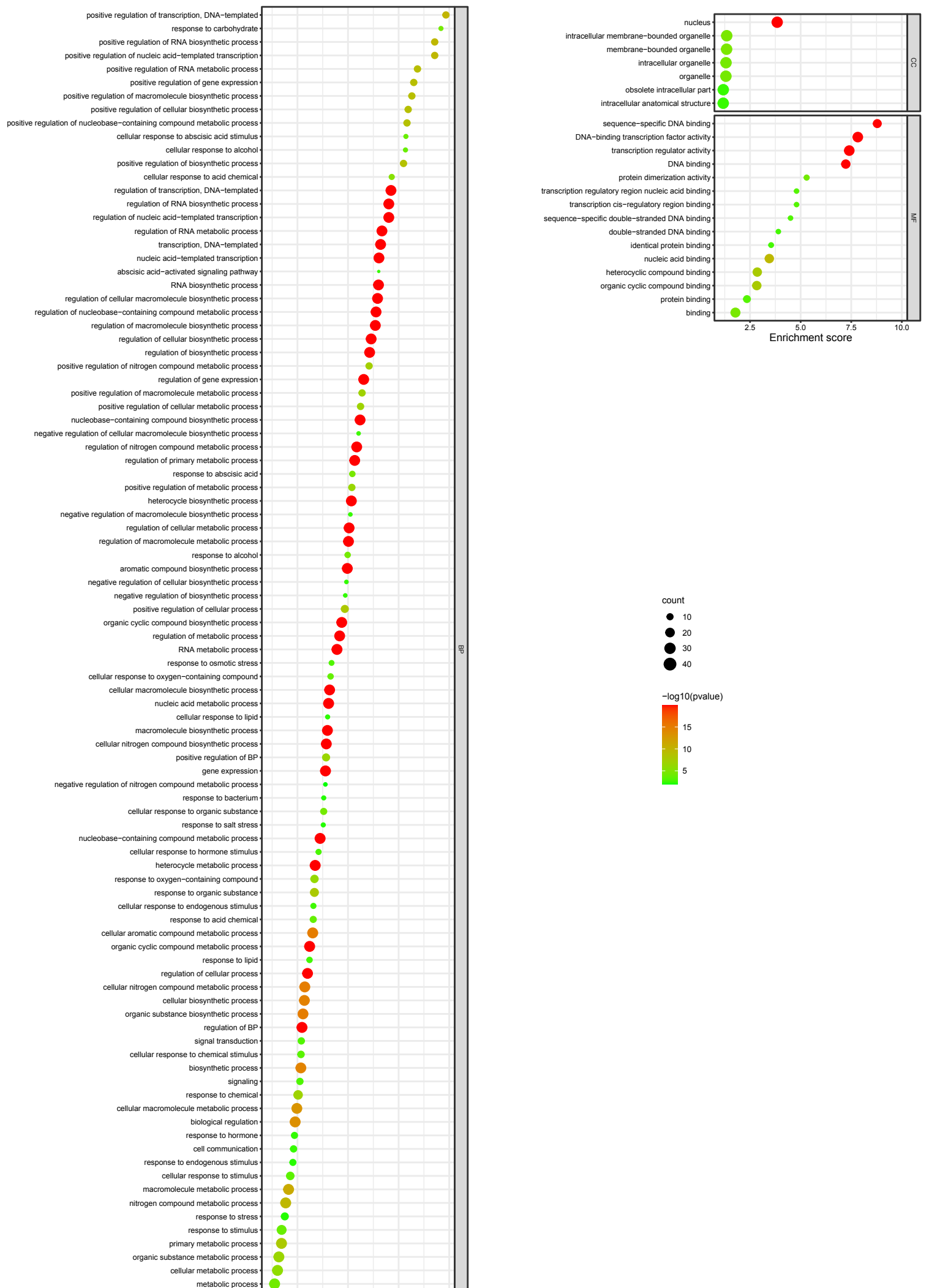

Fig. S6: Gene Ontology term distribution in *CpbZIP* genes.

Supplement: Supplementary file 6 — Additional file 6: Fig. S6. Gene Ontology term distribution in CpbZIP genes. [file 12864_2022_8978_MOESM6_ESM.pdf]
