# Supplementary material for: Genome-wide identification and analysis of bZIP gene family reveal their roles during development and drought stress in Wheel Wingnut (Cyclocarya paliurus)
Source: BMC Genomics. 2022 Nov 8;23:743. doi: 10.1186/s12864-022-08978-8 (PMC9641814; doi:10.1186/s12864-022-08978-8)
Supplement: Supplementary file 3 — Additional file 3: Fig. S3. Phylogenetic analysis of CpbZIP genes. The phylogenetic tree was constructed using IQ-tree with the maximum likelihood (ML) method and 1000 bootstrap replications. Black asterisks indicate putative duplicated genes. [file 12864_2022_8978_MOESM3_ESM.pdf]

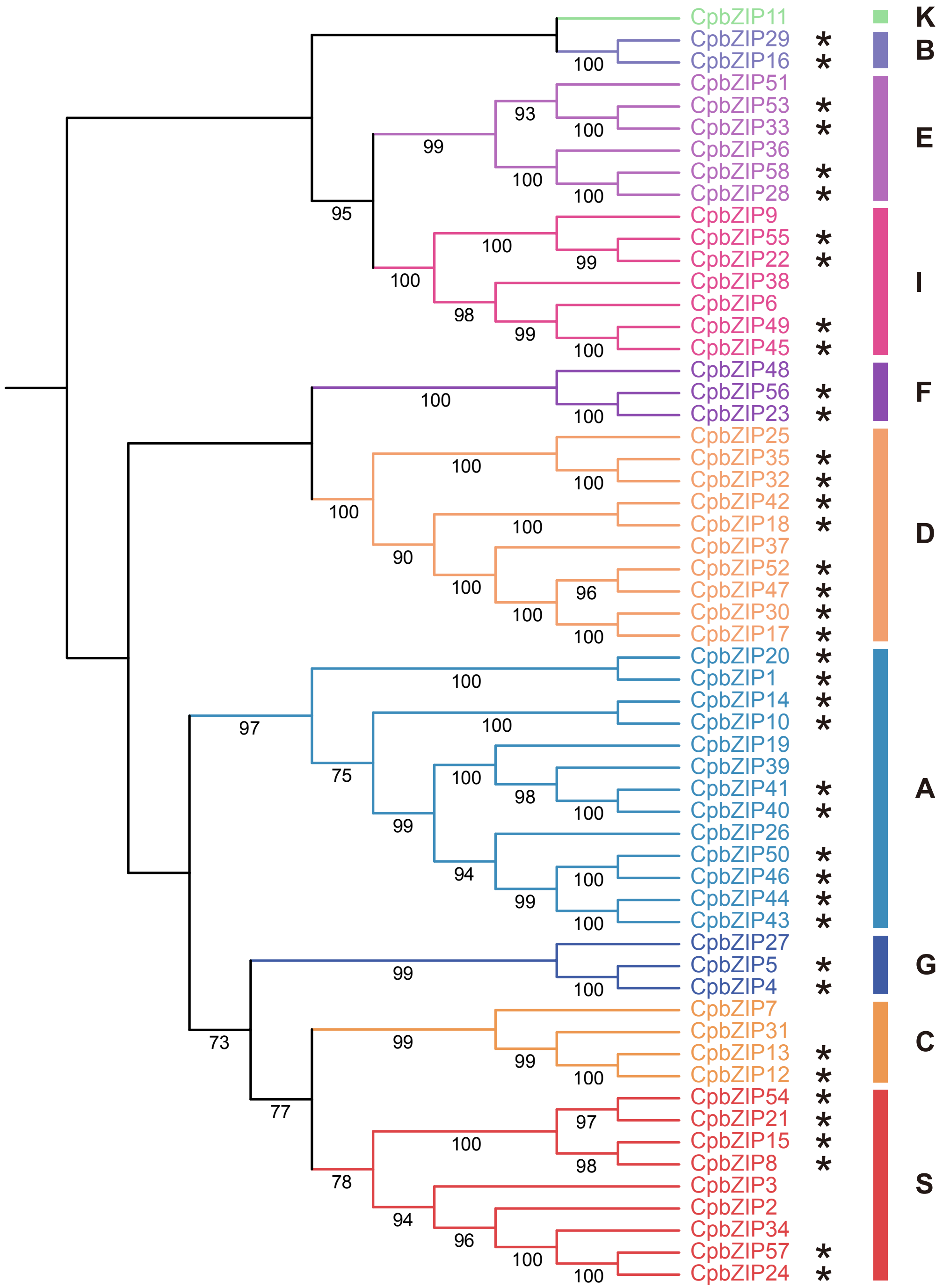

Fig. S3. Phylogenetic analysis of *CpbZIP* genes. The phylogenetic tree was constructed using IQ-tree with the maximum likelihood (ML) method and 1000 bootstrap replications. Black asterisks indicate putative duplicated genes.
