# Supplementary material for: Genome-wide identification and analysis of bZIP gene family reveal their roles during development and drought stress in Wheel Wingnut (Cyclocarya paliurus)
Source: BMC Genomics. 2022 Nov 8;23:743. doi: 10.1186/s12864-022-08978-8 (PMC9641814; doi:10.1186/s12864-022-08978-8)
Supplement: Supplementary file 5 — Additional file 5: Fig. S5. Distribution of intron numbers in CpbZIP genes in different groups according to the phylogenetic tree. [file 12864_2022_8978_MOESM5_ESM.pdf]

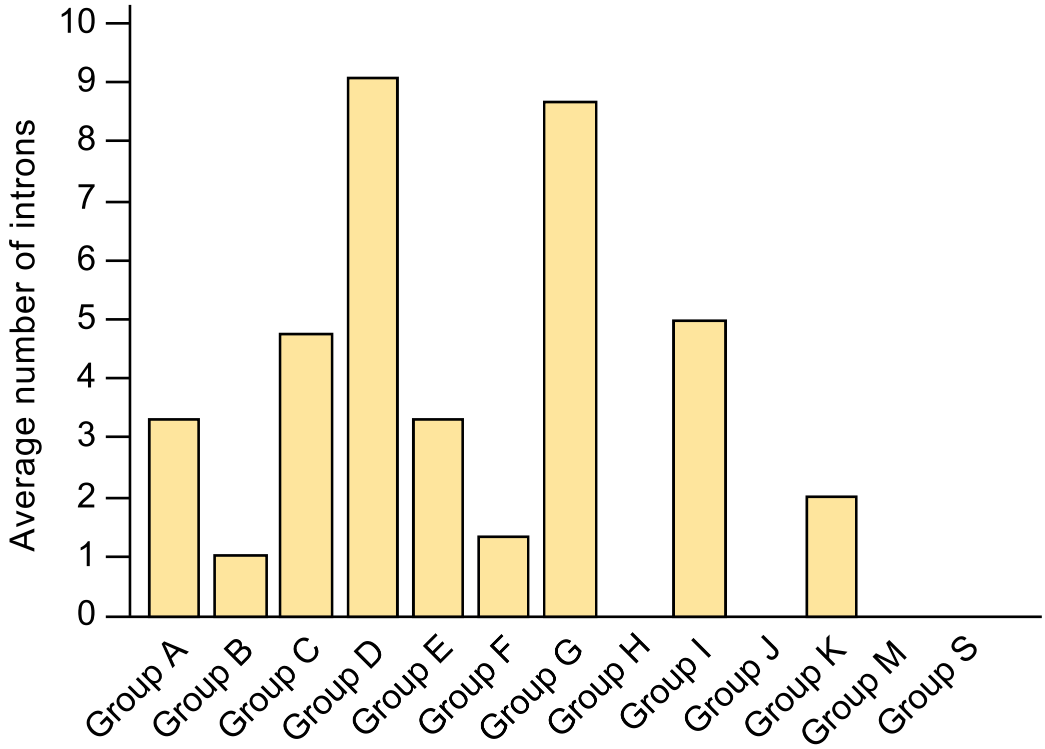

Fig. S5: Distribution of intron numbers in *CpbZIP* genes in different groups according to the phylogenetic tree.
