# Supplementary material for: Genome-wide identification and analysis of bZIP gene family reveal their roles during development and drought stress in Wheel Wingnut (Cyclocarya paliurus)
Source: BMC Genomics. 2022 Nov 8;23:743. doi: 10.1186/s12864-022-08978-8 (PMC9641814; doi:10.1186/s12864-022-08978-8)
Supplement: Supplementary file 7 — Additional file 7: Fig. S7. PCA plots displaying differentiation with respect to developmental stages and drought stress conditions based on CpbZIP expression patterns. [file 12864_2022_8978_MOESM7_ESM.pdf]

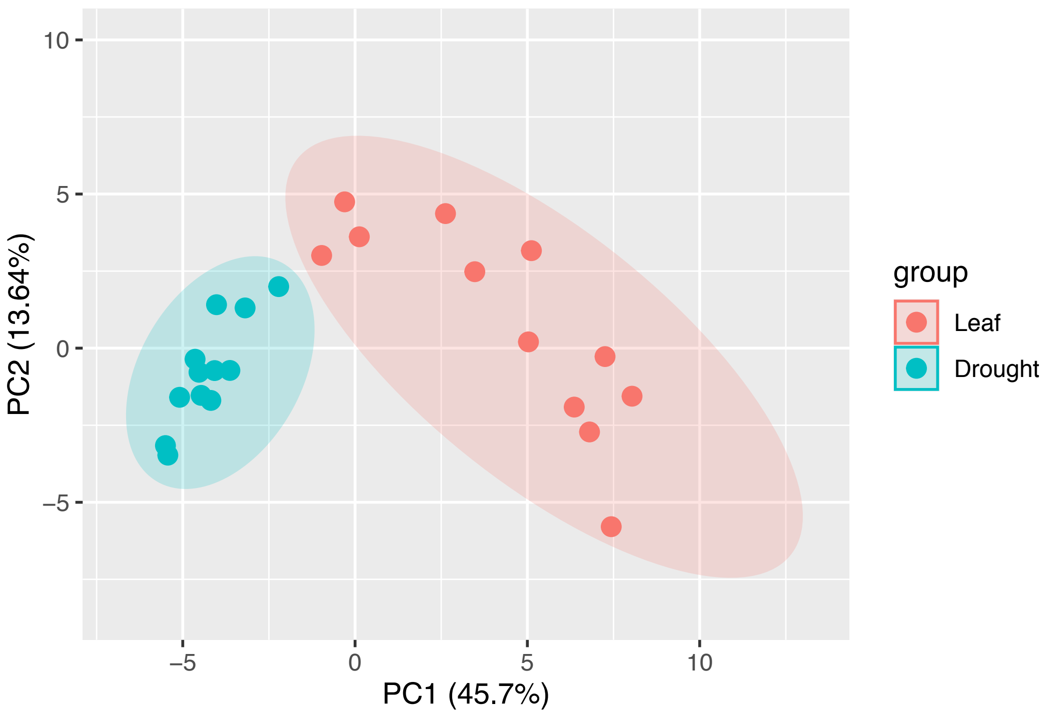

Fig. S7: PCA plots displaying differentiation with respect to developmental stages and drought stress conditions based on *CpbZIP* expression patterns.
